# NLRP3^+^ Macrophages: Central Inflammatory Nodes of Abdominal Aortic Aneurysm and Potential Cellular Target of Omega‐3 Polyunsaturated Fatty Acids

**DOI:** 10.1002/fsn3.72414

**Published:** 2026-11-10

**Authors:** Yuqi Wang, Hongrui Pan, Hechen Rong, Yu Zeng, Yinghong Li, Xiang Li, Zhian Liang, Jiaxin Wang, Lijian Fang, Peng Li, Xiangchen Dai

**Affiliations:** ^1^ Department of Vascular Surgery Tianjin Medical University General Hospital Tianjin China; ^2^ Tianjin Key Laboratory of Precise Vascular Reconstruction and Organ Function Repair Tianjin China; ^3^ Department of Vascular Surgery Tianjin General Surgery Institute Tianjin China; ^4^ Department of Infectious Diseases, The First Hospital of Tsinghua University, School of Clinical Medicine, Tsinghua Medicine Tsinghua University Beijing China; ^5^ Department of Radiation Oncology, Hainan General Hospital (Hainan Affiliated Hospital of Hainan Medical University) Haikou China; ^6^ Department of Vascular Surgery, Tianjin Union Medical Center The First Affiliated Hospital of Nankai University Tianjin China

**Keywords:** abdominal aortic aneurysm, inflammasome, macrophage, NLRP3, ω‐3 PUFA

## Abstract

Abdominal aortic aneurysm (AAA) is a fatal inflammatory vascular disease without effective clinical treatments. Dietary patterns regulate aortic inflammation, and Mediterranean diet‐derived omega‐3 polyunsaturated fatty acids (ω‐3 PUFA) exert anti‐inflammatory effects, yet their cellular targets in AAA remain unclear. We integrated three single‐cell RNA‐seq cohorts (12 AAA, 3 controls) and constructed a human aortic cell atlas with 64,258 cells. Pseudo‐bulk analysis and CoVarNet multicellular network analysis revealed a core remodeling axis: protective FOLR2^+^ resident macrophages were depleted, while a unique AAA‐enriched NLRP3^+^ inflammatory macrophage population (M08_Mph_NLRP3) formed the hub of pathological inflammatory networks. Three bulk transcriptomic cohorts and five complementary enrichment approaches verified this signature. An LPS/OX‐LDL/IL‐1β‐stimulated THP‐1 model recapitulated AAA macrophage pathological changes including NLRP3 inflammasome overactivation, enhanced proliferation and migration. Further functional tests demonstrated ω‐3 PUFA markedly blocked inflammasome cascade, reduced IL‐1β/IL‐18 release and reversed all macrophage pathological phenotypes. This study identifies M08_Mph_NLRP3 as the important inflammatory mediator of AAA and provides single‐cell and in vitro functional evidence supporting ω‐3 PUFA as a candidate nutritional agent targeting NLRP3‐driven vascular inflammation.

## Graphical abstract

We integrated three human aortic scRNA‐seq datasets to build an AAA cellular atlas and identified two key macrophage subsets. Homeostatic FOLR2^+^ macrophages are depleted in AAA, while NLRP3^+^ macrophages emerge as the core of inflammatory networks. In vitro, inflammatory stimuli induce pathological macrophage phenotypes. ω‐3 PUFA suppresses NLRP3 inflammasome activation and reverses macrophage dysfunction, alleviating AAA‐associated inflammation. (created by biorender.com).

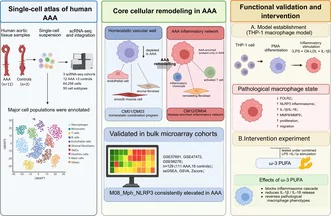

## 1 Introduction

Abdominal aortic aneurysm (AAA) represents a potentially fatal vascular disorder characterized by abnormal enlargement of the infrarenal segment of the aorta, affecting approximately 1%–2% of adults over 65 years of age and carrying a mortality rate exceeding 80% upon rupture (Guirguis‐Blake et al. 2019; Jiang et al. 2026; Shehab et al. 2026). Although surgical and endovascular repair methods have greatly improved clinical outcomes for AAA patients, no pharmacological therapy has demonstrated efficacy in halting AAA progression, reflecting a fundamental gap in our mechanistic understanding of the disease (Chao et al. 2024; Weaver et al. 2022). AAA is characterized by progressive degradation of the extracellular matrix, smooth muscle cell (SMC) loss, and chronic transmural inflammation, with the latter increasingly recognized as a central driver of aortic wall remodeling rather than a secondary consequence of structural failure (Liu et al. 2020; Márquez‐Sánchez and Koltsova 2022; Raffort et al. 2017; Shaikh et al. 2025; Weaver et al. 2022). Hence, clarifying how this inflammatory process operates at molecular and cellular levels lays the groundwork for novel targeted treatments.

Macrophages have long been implicated as key mediators of AAA pathogenesis (Li et al. 2022; Raffort et al. 2017; Wang and Murphy 2018; Wynn et al. 2013). Abundant macrophage infiltration is a hallmark of aneurysmal aortic tissue, and experimental depletion of macrophages attenuates AAA formation in murine models (Hou et al. 2024; Nahrendorf and Robbins 2025; Wu et al. 2024). However, macrophages are a functionally heterogeneous population encompassing homeostatic tissue‐resident subsets, inflammatory monocyte‐derived subsets, and disease‐associated transitional states, each with distinct transcriptional programs and functional roles (Bernier et al. 2025). Bulk transcriptomic studies have identified broad inflammatory signatures in AAA tissue, but the resolution afforded by such approaches is insufficient to attribute specific pathological programs to defined macrophage subpopulations (Le et al. 2024; Wu et al. 2022). In particular, the NLRP3 inflammasome—a multiprotein innate immune complex that may promote proteolytic maturation and secretion of IL‐18 and IL‐1β—has been implicated in AAA through immunohistochemical and pharmacological studies, yet the precise cellular identity of NLRP3‐expressing macrophages within the AAA microenvironment, and their relationship to other cell types in the aortic wall, has not been defined at single‐cell resolution (Huang et al. 2025; Ke et al. 2026).

Single‐cell RNA sequencing (scRNA‐seq) has transformed our ability to characterize cellular heterogeneity in complex tissues and has been applied to AAA in several recent studies, revealing diverse immune and stromal cell populations in the aneurysmal aortic wall (Weng et al. 2022; Yuan et al. 2024; Zhao et al. 2025). Nevertheless, existing single‐cell studies of AAA have largely focused on cataloging cell types and their differential abundance rather than investigating how individual cell subtypes interact to form coordinated multicellular programs. This distinction is biologically important: The pathological consequences of macrophage activation in AAA are likely to depend not only on the intrinsic properties of individual macrophage subsets but also on their coordination with other cell types including fibroblasts, SMCs, endothelial cells, and lymphocytes (Filiberto et al. 2022; Yao et al. 2024; Zhao et al. 2025). Multicellular coordination analysis frameworks that move beyond single‐cell cataloging to characterize the covariation of cell type frequencies across samples offer a powerful complementary approach to address this question (Agrawal et al. 2024; Shi et al. 2025).

Epidemiological and preclinical data have confirmed that dietary patterns are vital regulators of aortic wall inflammation and NLRP3 inflammasome activity (Yin et al. 2022; Zheng et al. 2022). Western diets high in saturated fats, cholesterol, and oxidized lipids trigger dyslipidaemia and elevate the dietary inflammatory index (DII), whereas Mediterranean diets abundant in omega‐3 polyunsaturated fatty acids (ω‐3 PUFA) suppress inflammasome signaling and relieve vascular inflammation (Yin et al. 2022). Large cohort studies show circulating ω‐3 PUFA can serve as reliable nutritional biomarkers negatively associated with AAA progression (Amirsardari et al. 2024). However, the specific mechanisms by which ω‐3 PUFA restrains aneurysmal inflammation remain unclear. Accordingly, we conducted multiple in vitro functional assays to dissect the regulatory role of ω‐3 PUFA in AAA pathological phenotypes.

Here, we integrated scRNA‐seq data from three AAA datasets comprising 64,258 cells from 12 AAA patients and three healthy controls, constructing a comprehensive multi‐lineage cellular atlas of AAA at single‐cell resolution. Within the myeloid compartment, we identified a discrete NLRP3^+^ inflammatory macrophage population (M08_Mph_NLRP3) that was present in AAA tissue within the present dataset, alongside a marked depletion of homeostatic FOLR2^+^ tissue‐resident macrophages (M09_Mph_FOLR2). To investigate the systems‐level implications of these single‐cell findings, we applied CoVarNet—a recently developed multicellular coordination network framework—to characterize how these macrophage shifts relate to broader changes in multicellular organization in AAA. Finally, we validated the disease relevance of the M08_Mph_NLRP3 signature through signature‐based deconvolution of three bulk transcriptomic microarray datasets comprising 129 samples across five complementary enrichment approaches. Together, our findings identify the reciprocal loss of homeostatic FOLR2^+^ macrophages and emergence of NLRP3^+^ inflammatory macrophages as a defining axis of cellular remodeling in AAA, and establish M08_Mph_NLRP3 as the central node of a disease‐enriched multicellular inflammatory network with implications for potential cellular targeting of the NLRP3 inflammasome pathway in AAA.

## 2 Methods

### 2.1 Data Acquisition and Preprocessing

Raw scRNA‐seq data were obtained from three publicly available AAA datasets submitted in the Gene Expression Omnibus (GEO): GSE166676, GSE226492, and GSE237230. Each dataset was processed independently prior to integration. For each sample, gene expression matrices were loaded using the Seurat (v4) framework. Cells were retained if they satisfied the following quality control criteria: number of detected genes between 200 and 6000, total UMI counts between 500 and 50,000, and mitochondrial gene percentage below 15%. Doublets were identified and removed using scDblFinder, with the expected doublet rate set according to the estimated cell recovery per sample. A total of 64,258 high‐quality cells were retained across 15 samples (12 AAA, 3 Control) for downstream analysis.

### 2.2 Data Integration and Dimensionality Reduction

Gene expression counts from all samples were merged into a single Seurat object. All sample expression matrices were integrated into one unified Seurat object. We conducted library normalization with a scaling factor of 10,000 and applied logarithmic transformation afterwards. The FindVariableFeatures function based on the vst algorithm was applied to select the top 2000 variable genes. Data were scaled and centered using ScaleData, with mitochondrial percentage regressed out as a technical covariate. Principal component analysis (PCA) was performed on the scaled data using the top 2000 variable features. To correct for inter‐dataset batch effects, Harmony was applied to the PCA embedding using dataset of origin as the batch variable, with a maximum of 20 iterations. The corrected low‐dimensional embedding was used for all subsequent clustering and visualization steps. Uniform Manifold Approximation and Projection (UMAP) was computed using the first 20 Harmony‐corrected principal components with n.neighbors = 30 and min.dist = 0.3.

### 2.3 Cell Clustering and Annotation

A shared nearest neighbor (SNN) graph was constructed using the FindNeighbors function based on the first 20 Harmony‐corrected dimensions. Unsupervised clustering was performed using the FindClusters function with a resolution of 0.3, yielding 23 initial clusters. We adopted distinct marker sets to classify cell populations: MS4A1, CD79A, and PAX5 marked B lymphocytes; CD3D, CD3E, and IL7R indicated T cell subsets; NKG7, GNLY, and KLRF1 served as signatures for ILC/NK cells. For myeloid, endothelial, and stromal cells, we used CD68/C1QA/LYZ, PECAM1/VWF/CDH5, and DCN/LUM/COL1A1, respectively. Clusters with unambiguous contamination signatures (AGR2^+^ epithelial cells, HBD^+^ erythrocytes) or insufficient cell numbers were excluded. Finally, we sorted all valid cells into six primary lineages: B cells, CD4^+^ T cells, ILC/NK cells, myeloid cells, endothelial cells, and stromal cells.

We isolated all primary cell lineages and ran independent re‐clustering steps to gain higher resolution subtype information. All samples went through identical analytical steps: variable feature filtering, data scaling, PCA computation, Harmony adjustment and UMAP plotting. We further optimized clustering resolutions for every lineage, ensuring the final cell populations conformed to biologically verified subtypes from authoritative annotation databases. Subtype identities were assigned by cross‐referencing top marker genes from FindAllMarkers (Wilcoxon rank‐sum test, minimum expressed fraction 0.25, log2 fold‐change threshold 0.4) against canonical marker gene sets derived from published single‐cell atlases. Contaminating non‐lineage cells identified during sub‐clustering were reassigned to their correct major lineage or excluded, and their subtype labels were resolved accordingly. A total of 50 cell subtypes were defined across the six major lineages. Subtype labels followed the naming convention of the pan‐tissue cell atlas described by Qi et al. to ensure compatibility with the CoVarNet reference network.

### 2.4 Pseudo‐Bulk Differential Expression Analysis

To identify transcriptional differences between AAA and control conditions within each cell type, pseudo‐bulk differential expression analysis was performed using DESeq2. For each cell subtype, cells were aggregated by sample to generate pseudo‐bulk count profiles. Samples with fewer than 10 cells for a given subtype were excluded from that subtype's analysis, and subtypes with fewer than three valid samples in either condition were not tested. Raw counts were used as input to DESeqDataSetFromMatrix with condition as the design variable. Genes with counts per million below 1 in all samples were filtered prior to analysis. Differentially expressed genes (DEGs) were defined by a false discovery rate (FDR) below 0.2 and absolute log2 fold‐change > 0.5. The relaxed FDR threshold was applied given the limited number of control samples (*n* = 3). Major lineage‐level pseudo‐bulk analysis was performed using the same pipeline applied at the majorCluster level.

### 2.5 Gene Ontology Enrichment and Gene Set Enrichment Analysis

Overrepresentation analysis (ORA) of Gene Ontology Biological Process (GO BP) terms was performed using the enrichGO function from the clusterProfiler package, with the full set of tested genes as background, a *p*‐value cutoff of 0.05, and a *q*‐value cutoff of 0.2. Gene set enrichment analysis (GSEA) was performed using the gseGO function from clusterProfiler, with genes ranked by log2 fold‐change derived from pseudo‐bulk DESeq2 results. Minimum and maximum gene set sizes were set to 10 and 500, respectively. Both analyses were performed against the *Homo sapiens* annotation database (org.Hs.eg.db) using gene symbols as input.

### 2.6 CoVarNet Multicellular Coordination Network Analysis

Multicellular coordination analysis was performed using the CoVarNet R package (Shi et al. 2025). Cell subtype frequency matrices were constructed using the freq_calculate and freq_normalize functions, with each sample represented as a vector of relative cell subtype frequencies. Samples with fewer than 200 total cells were excluded prior to frequency calculation, yielding a final analytical cohort of 15 samples (12 AAA, 3 Control).

For Recovery analysis, the reference network derived from the pan‐tissue human cell atlas was used, comprising 12 reference cellular modules (CMs) defined across 76 cell subtypes (Shi et al. 2025). We built a complete frequency matrix covering all 76 reference cell subtypes to maintain consistent dimensional matching. This step matched the 50 cell subsets uncovered within our dataset against standard reference labels, while any reference subtypes absent from our samples received a zero frequency value. We ran the sc_cm_recover function to map sample‐specific subtype frequency profiles onto the reference CM space, which generated CM activity metrics for every individual sample. Statistical disparities in CM activity values between AAA and control cohorts were evaluated via Wilcoxon rank‐sum testing.

For Discovery analysis, we performed nonnegative matrix factorization (NMF) on normalized frequency data of the 50 cell subtypes using the nsNMF algorithm from the R NMF library. The analysis included 30 random initialization replicates alongside a fixed random seed for reproducibility. We screened factorization ranks ranging from *K* = 2 to *K* = 12, selecting the optimal *K* value by comparing cophenetic correlation coefficients, dispersion metrics, and silhouette scores across all tested ranks. A rank of *K* = 6 was chosen as the final parameter, given the cophenetic correlation coefficient exhibited far greater stability at this rank compared to neighboring *K* values. We profiled the six extracted Discovery Modules (DMs) through weights from the output basis matrix, and relied on the top 10 most enriched cell subtypes within each DM to conduct downstream biological interpretation. Pairwise Pearson correlation calculations across all specimens were used to build covariation networks of cell subsets for every DM; only correlation pairs meeting an *r* > 0.20 cutoff and predefined specificity score limits were kept as network edges. All network plots were rendered with the igraph R package. To combine outputs from compartment recovery and discovery decomposition pipelines, we calculated Spearman correlation linking CM activity scores and DM activity values across all 15 study samples.

### 2.7 Bulk Transcriptomic Data Acquisition and Preprocessing

Three publicly available microarray datasets were obtained from GEO: GSE57691 (Illumina HumanHT‐12 V4, AAA = 49, Control = 10), GSE47472 (Illumina HumanHT‐12 V4, AAA = 14, Control = 8), and GSE98278 (Illumina HumanHT‐12 V4, AAA = 48). Raw expression values were processed as follows: probe‐level intensities were background‐corrected by setting negative values to 1 and log2‐transformed. Quantile normalization was applied across all samples using the normalizeBetweenArrays function from the limma package. Probe‐to‐gene symbol mapping was performed using the illuminaHumanv4.db annotation package, retaining the probe with the highest mean expression per gene in cases of multiple probes mapping to the same symbol. Genes not expressed above background in any sample were removed, yielding a final matrix of 19,391 genes across 129 samples. Inter‐dataset batch effects were corrected using ComBat from the sva package, with dataset of origin as the batch variable and condition (AAA vs. Control) as a protected biological covariate.

### 2.8 Cell Type Signature‐Based Deconvolution

Signature gene sets for 19 cell subtypes were derived from pseudo‐bulk DESeq2 marker analysis of the single‐cell dataset, selecting genes significantly upregulated in each subtype relative to all other subtypes (FDR < 0.05, log2FC > 1). Five complementary enrichment approaches were applied to the batch‐corrected bulk expression matrix: single‐sample gene set enrichment analysis (ssGSEA) implemented via the GSVA package; Gene Set Variation Analysis (GSVA) using the gsva function with default parameters; *Z*‐score‐based enrichment (Zscore); mean expression scoring (MeanExpr); and weighted mean expression (WMean). For each method, enrichment scores were computed per sample per signature, yielding a 129‐sample × 19‐signature score matrix. Statistical significance of score differences between AAA and control groups was assessed using the Wilcoxon rank‐sum test for each method independently. A signature was considered consistently significant if it reached *p* < 0.05 in three or more of the five methods. The number of methods reaching significance (*p* < 0.05) per signature was used as a consensus metric for cross‐method robustness. Method‐level concordance was assessed by computing pairwise Spearman correlations between deconvolution score vectors across methods for each signature.

### 2.9 Statistical Analysis

All statistical analyses were performed in R (version 4.3). Group comparisons were performed using the Wilcoxon rank‐sum test unless otherwise stated. Multiple testing correction was applied using the Benjamini–Hochberg method where applicable. Spearman correlation coefficients were computed using the cor function with method = “spearman”. All visualizations were generated using ggplot2, pheatmap, and igraph packages. Significance thresholds are indicated in figures as follows: *p < 0.05, **p < 0.01, ***p < 0.001, ****p < 0.0001.

### 2.10 ELISA

According to manufacturer's instructions, human interleukin1β (IL‐1β) and human interleukin‐18 (IL‐18) from THP‐1‐derived macrophages were assessed using ELISA kits (Elabscience Biotechnology Co. Ltd).

### 2.11 Western Blot (WB)

Total proteins were isolated from cultured cells and tissue specimens with RIPA lysis buffer (Beyotime) containing 1× protease and phosphatase inhibitor cocktail (Beyotime). After sufficient lysis, the mixtures were centrifuged at 12,000 rpm for 15 min at 4°C, and the resulting supernatants were harvested for protein concentration determination using a BCA Protein Quantification Kit (Beyotime). Equivalent quantities of protein samples were combined with 5× SDS‐PAGE loading buffer, heated at 100°C for 10 min to achieve full denaturation, and then subjected to sodium dodecyl sulfate‐polyacrylamide gel electrophoresis. Following electrophoretic separation, proteins were transferred onto PVDF membranes, which were subsequently blocked with 5% bovine serum albumin (BSA) at room temperature for 1 h. The membranes were then incubated with corresponding primary antibodies at 4°C overnight, and further incubated with horseradish peroxidase (HRP)‐conjugated secondary antibodies (BOSTER, 1:8000 dilution) for 1 h at room temperature. Protein bands were detected and imaged using a Bio‐Rad Universal Hood III imaging system. A detailed list of all antibodies applied in the present study is provided in Table S1.

### 2.12 RNA Isolation and qRT‐PCR

For the assessment of mRNA expression levels, a QIAGEN RNeasy mini kit (Cat #74104) was utilized for RNA extraction according to the manufacturer's instructions. Quantitative real‐time PCR was performed using SYBR Green PCR Master Mix (Cat #4309155) on a QuantStudio 12 K Flex instrument (Table S2).

### 2.13 THP‐1 Cell Culture and Inflammatory Model Establishment

THP‐1 cells were cultured in RPMI‐1640 medium supplemented with 10% fetal bovine serum, 1% penicillin–streptomycin mixture, and 0.05 mM β‐mercaptoethanol. Cells were kept in a humidified incubator at 37°C with 5% CO_2_ throughout routine maintenance. To induce differentiation toward a macrophage‐like phenotype, THP‐1 cells were seeded at a density of 1 × 10^6^ cells/mL and treated with 100 ng/mL phorbol 12‐myristate 13‐acetate (PMA) for a continuous 48‐h period. Following the differentiation period, cells were rinsed twice with PBS. For the inflammatory model groups, several treatments were applied: 50 μg/mL OX‐LDL treatment for 24 h to simulate lipid oxidative damage; 10 ng/mL IL‐1β treatment for 24 h to mimic NLRP3 downstream inflammation;10 ng/mL LPS treatment for 24 h to simulate monocyte infiltration and combined administration of the above agents to generate an inflammatory macrophage phenotype associated with the transcriptional features observed in the single‐cell analysis. After completing the 24 h stimulation period containing recombinant IL‐1β, culture medium was fully aspirated, cells were washed twice with PBS, and fresh stimulus‐free complete medium was added for subsequent incubation. The normal control group received no treatment. Cells received 10 μM MCC950 (PZ028, Sigma) 1 h before stimulation to inhibit NLRP3 inflammasomes. Cells received 20 μM ω‐3 PUFA 24 h before stimulation to inhibit NLRP3 inflammasomes. ω‐3 PUFA, eicosapentaenoic acid (EPA, Sigma‐Aldrich, Cat. No. E2011, ≥ 99% purity) and docosahexaenoic acid (DHA, Sigma‐Aldrich, Cat. No. D2534, ≥ 98% purity), were used at a 1:1 M ratio. Stock solutions (100 mM) of EPA and DHA were separately prepared in anhydrous ethanol under nitrogen protection, aliquoted, and stored at −80°C in the dark. Fatty‐acid‐free BSA (Sigma‐Aldrich, Cat. No. A6003) was used to generate fatty acid‐BSA complexes at a 3:1 fatty acid to BSA molar ratio by gentle incubation at 37°C for 1 h. For cell treatment, the complex was diluted into culture medium to a final total ω‐3 PUFA concentration of 20 μM. Corresponding vehicle control medium contained identical concentrations of fatty acid free BSA and ethanol without added EPA/DHA.

### 2.14 Transwell Assays

For Transwell migration assays, 8.0 μm pore Transwell inserts were applied. THP‐1‐derived macrophages were resuspended in serum‐free RPMI‐1640 medium and seeded into the upper chambers at 2 × 10^5^ cells/well. The lower chambers were supplemented with complete medium containing 10% fetal bovine serum. Following 24 h incubation at 37°C and 5% CO_2_, non‐migrated cells on the upper membrane were removed. Migrated cells were fixed with 4% paraformaldehyde, stained with 0.1% crystal violet, and imaged under a light microscope. Migrated cells in several random fields per sample were quantified for statistical analysis. All experiments were performed with three independent biological replicates.

### 2.15 EdU Assays

Cell proliferation was detected using a 5‐ethynyl‐2′‐deoxyuridine (EdU) staining kit according to the manufacturer's protocols. EdU working solution was added to the culture medium and incubated for 2 h at 37°C with 5% CO_2_. Subsequently, cells were fixed with 4% paraformaldehyde for 15 min and permeabilized with 0.5% Triton X‐100 for 10 min at room temperature. After washing with PBS, cells were incubated with Click‐iT reaction solution for 30 min in the dark. Cell nuclei were counterstained with Hoechst 33342. Fluorescence images were captured using a fluorescence microscope. All experiments were performed with three independent biological replicates.

## 3 Results

### 3.1 Integration of Multi‐Cohort Single‐Cell Transcriptomic Data Constructs a Comprehensive Cellular Atlas of AAA

To systematically characterize the cellular landscape of AAA, we integrated scRNA‐seq data from three public datasets (GSE166676, GSE226492, and GSE237230), comprising 12 AAA patients and three healthy controls. A total of 64,258 high‐quality cells were retained for downstream analysis after stringent quality control, doublet removal, and Harmony‐based batch correction (Figures 1A and 2A,B). Unsupervised clustering followed by marker‐gene‐guided annotation identified six major cell lineages: B cells (*n* = 9237), CD4^+^ T cells (*n* = 7920), innate lymphoid cells/NK cells (ILC, *n* = 948), myeloid cells (*n* = 18,293), endothelial cells (*n* = 6554), and stromal cells (*n* = 21,306) (Figure 1B,C). The identity of each major lineage was confirmed by canonical marker gene expression, including MS4A1 and CD79A for B cells, CD3D and CD4 for CD4^+^ T cells, NKG7 and GNLY for ILC/NK cells, C1QA and FOLR2 for myeloid cells, PECAM1 and VWF for endothelial cells, and DCN and MYH11 for stromal cells (Figure 1C,D).

**FIGURE 1:**
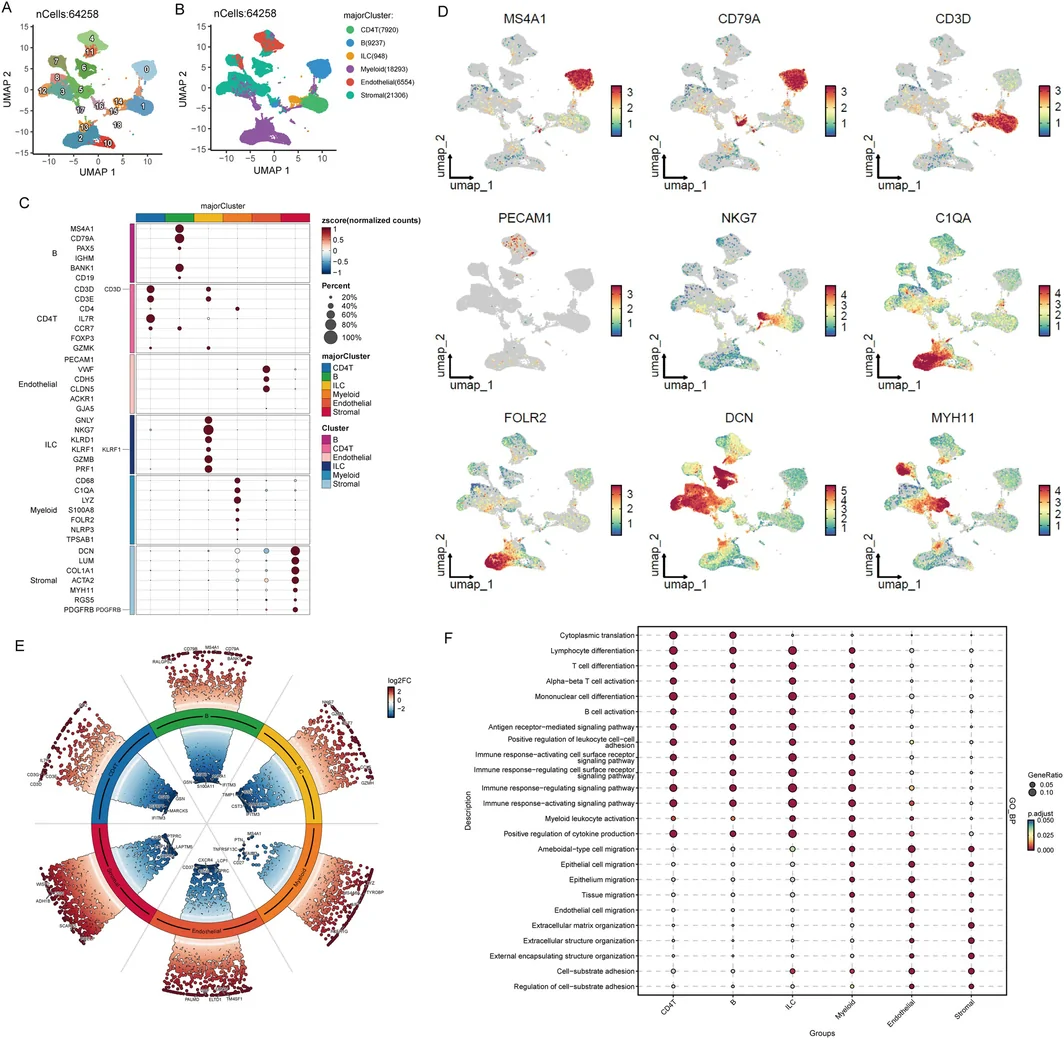
Single‐cell RNA‐seq profiling reveals the cellular landscape of human AAA tissues. (A, B) Uniform manifold approximation and projection (UMAP) visualization of 64,258 high‐quality single cells from AAA specimens, colored by (A) initial unsupervised clusters and (B) annotated major cell types. (C) Dot plot showing the expression of canonical marker genes across identified cell populations. Dot size indicates the percentage of cells expressing the gene, and color intensity represents average normalized expression level. (D) Feature plots on the UMAP embedding showing the distribution of key cell‐type‐specific markers, including MS4A1, CD79A, CD3D, PECAM1, NKG7, C1QA, FOLR2, DCN, and MYH11. (E) Circos plot visualizing the log_2_ fold change of signature gene expression across the major cell clusters. (F) GSEA results for biological processes in each cell population. Bubble size indicates adjusted *p*‐value, and color represents the normalized enrichment score (NES).

**FIGURE 2:**
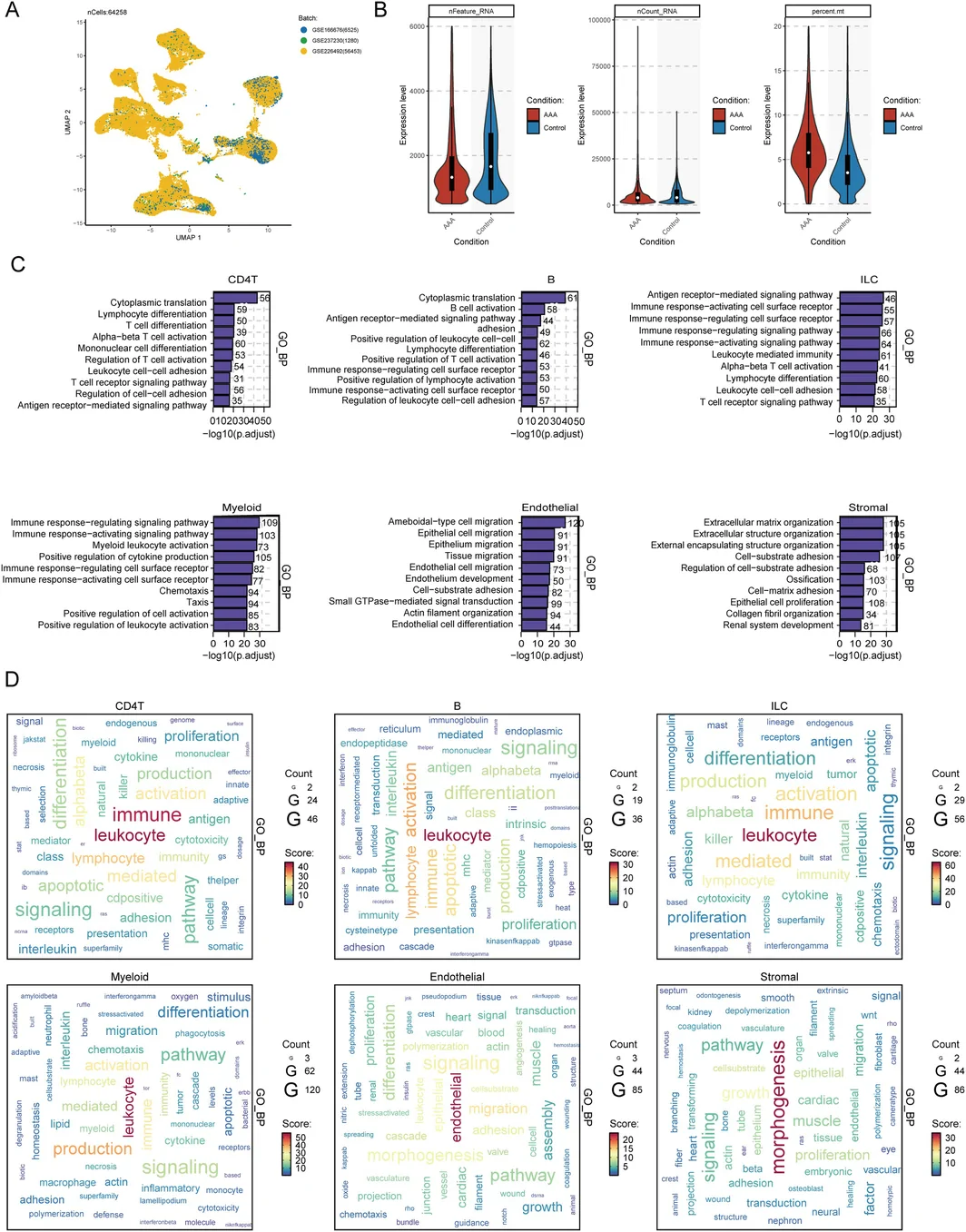
Functional enrichment analysis of major cell types identified in human AAA tissues. (A) Uniform manifold approximation and projection (UMAP) visualization (B) Violin plots showing the distribution of nFeature_RNA, nCount_RNA, and percent.mt in cells from AAA (red) and control (blue) groups. (C) GO‐BP enrichment analyses of myeloid cells, endothelial cells, and stromal cells, respectively. (D) Detailed GO‐BP terms and gene counts for CD4^+^ T cells, B cells and ILC, myeloid, endothelial, and stromal cells.

We carried out differential gene expression comparisons between AAA‐derived and control cells within each primary cell lineage, which uncovered extensive transcriptional reprogramming (Figure 1E). GO enrichment analysis of lineage‐specific DEGs highlighted distinct biological processes activated in each compartment: myeloid cells showed marked enrichment in immune response activation and cytokine production pathways, stromal cells in extracellular matrix organization and cell‐substrate adhesion, and endothelial cells in cell migration‐related processes (Figures 1F and 2C,D). These findings establish a multi‐lineage transcriptional atlas of AAA and provide a foundation for dissecting disease‐enriched cellular programs.

### 3.2 Disease‐Associated Cellular Remodeling Reveals the Emergence of NLRP3 ^+^ Inflammatory Macrophages and Depletion of FOLR2 ^+^ Tissue‐Resident Macrophages in AAA

To resolve cellular composition changes between AAA and healthy aortic tissue, we first quantified the proportion of each major lineage across all samples. In AAA tissues, B cell proportions were notably elevated, while stromal and endothelial cell proportions were substantially reduced; notably, the overall proportion of myeloid cells remained broadly comparable between AAA and control groups (Figure 3A,B). However, myeloid cells are well established as a key inflammation‐associated macrophage population correlated with AAA pathogenesis, raising the question of whether disease‐relevant changes are driven not by global myeloid abundance but by shifts in the composition and transcriptional states of specific myeloid subtypes. To address this, we performed pseudo‐bulk differential expression analysis across all major lineages. This revealed that myeloid cells harbored by far the greatest number of DEGs between AAA and control conditions, followed by endothelial cells—a pattern fully consistent with the inflammatory and vascular remodeling mechanisms underlying AAA (Figure 3C). These findings motivated a focused investigation of myeloid cell heterogeneity as the primary analytical priority in subsequent analyses. GO enrichment analysis of myeloid DEGs upregulated in AAA revealed activation of immune response signaling, enhanced cytokine synthesis as well as the activation of myeloid leukocyte, whereas genes elevated in controls were enriched for angiogenesis and tissue development processes (Figure 3D). In the stromal compartment, AAA‐upregulated genes were associated with collagen metabolic processes and extracellular matrix organization, while control‐enriched genes reflected normal vascular SMC maintenance (Figure 3E).

**FIGURE 3:**
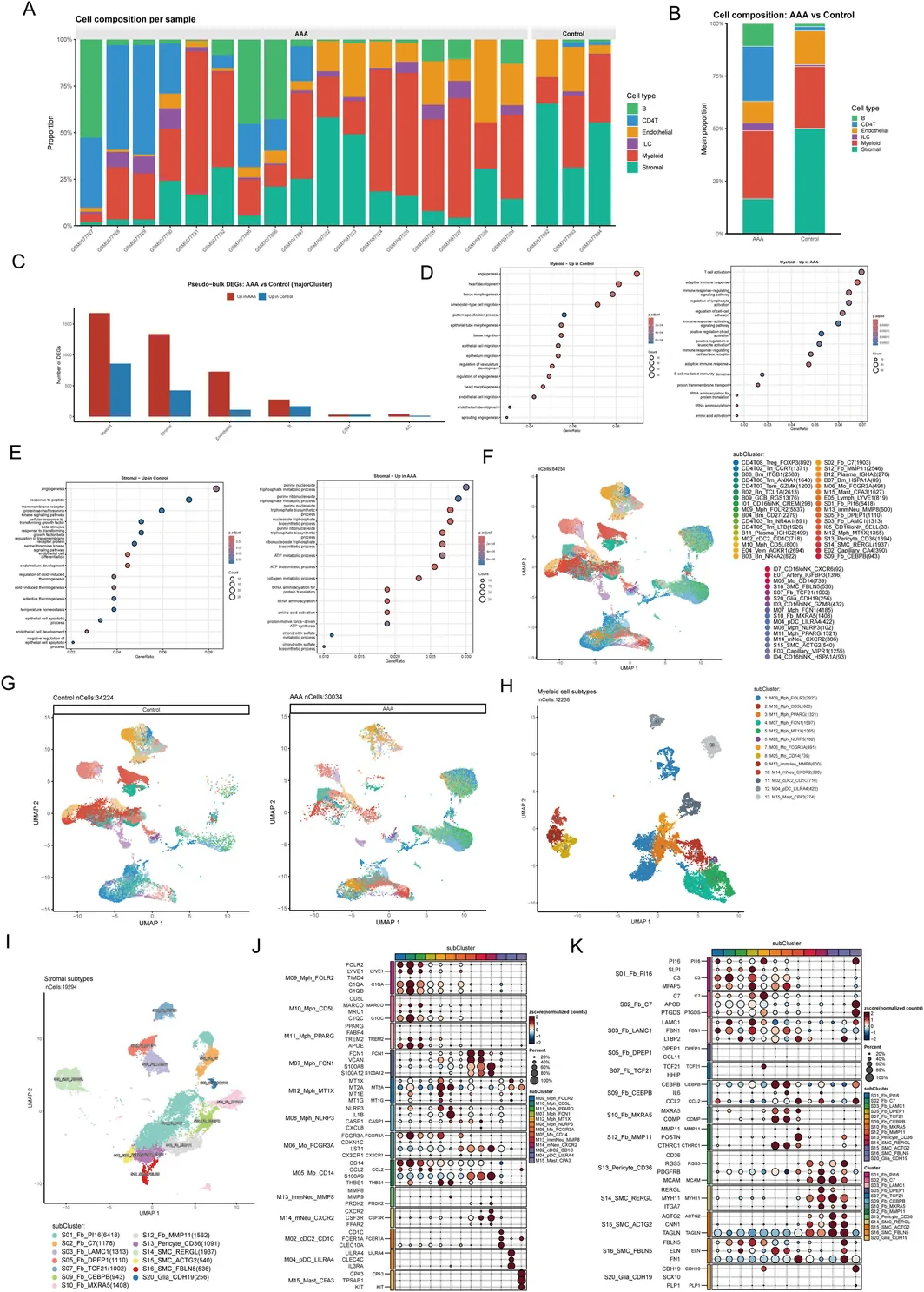
Cellular composition heterogeneity and functional differences between AAA and control tissues. (A) Overview of cellular composition distribution in all samples. (B) Comparative analysis of cellular composition between AAA and control groups. (C) Differential characterization of cell subpopulations and gene expression profiles. (D, E) Functional enrichment analysis of distinct cell subsets. (F) UMAP visualization of all cells colored by fine‐grained cell subclusters. (G) UMAP visualization showing the distribution of cells from control (left, *n* = 34,224 cells) and AAA (right, *n* = 30,034 cells) samples. (H) UMAP visualization of myeloid cell subpopulations (*n* = 12,238 cells), colored by subclusters with corresponding annotations on the right. (I) UMAP plot of all stromal subpopulations isolated from aortic tissues (*n* = 19,294 cells), with individual stromal subtypes annotated and colored as shown in the lower left legend. (J) Dot heatmap displaying the top marker genes defining each myeloid subcluster. (K) Dot heatmap illustrating signature marker genes for all stromal cell subclusters.

To achieve higher resolution, we performed sub‐clustering within each major lineage, ultimately identifying 50 cell subtypes across the entire dataset (Figure 3F,G, Figure S1). Within the myeloid compartment (*n* = 12,238 cells), we characterized 13 distinct subtypes based on canonical marker gene expression (Figure 3H,J). Tissue‐resident macrophages expressing FOLR2, LYVE1, TIMD4, and C1QA were designated M09_Mph_FOLR2 (*n* = 2923), consistent with previously described homeostatic tissue‐resident macrophages. We discovered a rare discrete macrophage cluster positive for NLRP3, IL1B, and CASP1 that solely existed in AAA tissues (*n* = 102, AAA = 102, Control = 0); we labeled it M08_Mph_NLRP3 as a AAA‐enriched inflammatory macrophage subtype. Additional myeloid subtypes including FCN1^+^ inflammatory macrophages (M07_Mph_FCN1), MT1X^+^ stress‐response macrophages (M12_Mph_MT1X), PPARG^+^ lipid‐associated macrophages (M11_Mph_PPARG), classical monocytes (M05_Mo_CD14), nonclassical monocytes (M06_Mo_FCGR3A), immature neutrophils (M13_immNeu_MMP8), mature neutrophils (M14_mNeu_CXCR2), plasmacytoid dendritic cells (M04_pDC_LILRA4), conventional dendritic cells (M02_cDC2_CD1C), and mast cells (M15_Mast_CPA3) were also identified.

Notably, M09_Mph_FOLR2 and M08_Mph_NLRP3 displayed opposite correlations with aneurysm status. The M09_Mph_FOLR2 subset was highly abundant within control tissues, with 2232 resident cells versus merely 691 cells found in AAA specimens; in sharp contrast, M08_Mph_NLRP3 cells could only be observed in aneurysm lesions (Figure 3H). We analyzed a total of 19,294 stromal single cells and distinguished 16 distinct subpopulations from this compartment. These subgroups covered 12 fibroblast clusters marked S01 to S12, three separate SMC subsets (S14_SMC_RERGL, S15_SMC_ACTG2, S16_SMC_FBLN5), CD36‐expressing pericytes labeled S13_Pericyte_CD36, alongside glial cell populations annotated as S20_Glia_CDH19 (Figure 3I,K). Together, our findings uncovered the full cellular heterogeneity of AAA at the single‐cell level. We also confirmed that the opposing changes between homeostatic FOLR2^+^ macrophages and disease‐related NLRP3^+^ inflammatory populations represent a key pathological characteristic of this vascular disorder.

### 3.3 CoVarNet Analysis Reveals Disruption of a Homeostatic Multicellular Module and Emergence of an NLRP3‐Driven Disease‐Enriched Multicellular Network in AAA

The identification of opposing shifts in M09_Mph_FOLR2 and M08_Mph_NLRP3 at the single‐cell level prompted us to investigate whether these changes reflect broader disruptions in multicellular coordination. We applied CoVarNet, a computational framework that identifies covarying cell subtype frequency modules across samples, to systematically characterize multicellular coordination programs in AAA. The analysis was conducted in two complementary strategies: a Recovery analysis, which projects our data onto a reference network of 12 CMs derived from a large‐scale pan‐tissue atlas of normal human tissues, and a Discovery analysis, which de novo identifies disease‐relevant multicellular modules from our AAA dataset.

In the Recovery analysis, the activity of all 12 reference CMs was quantified across the 15 samples and compared between AAA and control groups (Figure 4A, Figure S2A). Among all CMs, CM12 showed a trend toward higher activity in AAA compared to controls (*p* = 0.030), while CM01 exhibited a trend toward reduced activity in AAA, though no significant differences were observed (*p* = 0.071) (Figure 4B). Inspection of the CM12 network revealed that M08_Mph_NLRP3 was a central node, co‐occurring with inflammatory monocytes, activated T cells, and fibroblasts, suggesting that NLRP3^+^ macrophages participate in a coordinated multicellular inflammatory program in AAA (Figure 4C). In contrast, the CM01 network was anchored by M09_Mph_FOLR2 together with vascular wall‐associated cell types including stromal fibroblasts and endothelial cells, consistent with a homeostatic tissue‐maintenance function (Figure 4C, Figure S2B,C). These results indicate that the reference homeostatic multicellular module is attenuated in AAA while a disease‐associated module centered on NLRP3^+^ macrophages becomes activated.

**FIGURE 4:**
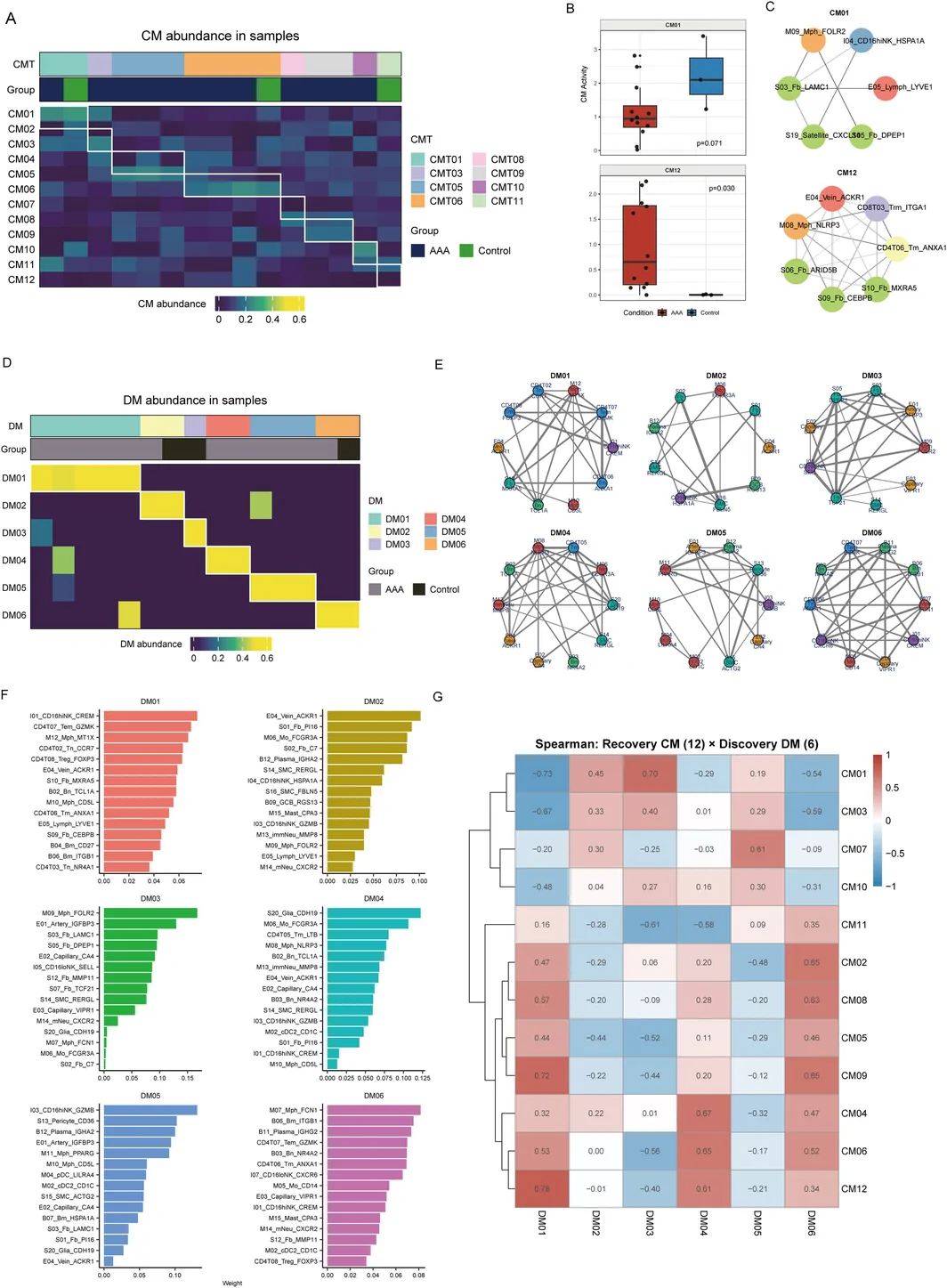
Abundance distribution of CM and DM subsets and their correlation analysis in AAA and control groups. (A) Abundance of CM subsets in each sample. (B) Comparison of CM subset abundance between AAA and control groups. (C) Multicellular covariation network of two representative cellular microenvironment modules (CM01 and CM12) in AAA tissues. (D) Abundance of DM subsets in each sample. (E) DM subset abundance. (F) Weighted contribution of cell subpopulations to six disease‐associated cellular modules (DM01–DM06) in AAA tissues. (G) Spearman correlation analysis between recovery CM (12 subsets) and discovery DM (six subsets).

To complement the Recovery analysis and identify AAA‐enriched multicellular coordination patterns not captured by the reference network, we performed a Discovery analysis using NMF on the cell subtype frequency matrix derived from our dataset. After evaluating multiple quantitative metrics for NMF rank screening, we fixed the factorization rank at *K* = 6, which generated six distinct DMs (Figure S3A). We quantified module activity values for all DMs across every experimental sample: DM01, DM04, and DM05 were predominantly active in AAA samples, whereas DM03 was enriched in control samples (Figure 4D). Examination of the cell subtype composition of each DM further underscored this dichotomy (Figure 4E,F, Figure S3B,C). DM03, the control‐enriched module, was anchored by M09_Mph_FOLR2 together with arterial endothelial cells (E01_Artery_IGFBP3), stromal fibroblasts, and SMCs, collectively representing a homeostatic vascular wall coordination program. In contrast, DM04, one of the AAA‐dominant modules, was characterized by M08_Mph_NLRP3 as a top‐weighted component alongside FCN1^+^ inflammatory macrophages, activated T cells, and remodeling fibroblasts, delineating a disease‐enriched inflammatory multicellular network.

We computed Spearman correlations between the activity scores of the 12 reference CMs and the six de novo DMs across all samples to integrate the Recovery and Discovery results and assess their mutual consistency (Figure 4G). We also detected a marked positive linear association between activity scores of CM01 and DM03 (*r* = 0.70); such consistent correlation further validates that both analyses converge on the same homeostatic program anchored by FOLR2^+^ macrophages. Conversely, CM12 activity showed strong positive correlations with DM01 (*r* = 0.78) and DM04 (*r* = 0.61), both of which are AAA‐dominant modules containing M08_Mph_NLRP3 as a key component. This cross‐framework concordance provides robust evidence that the reciprocal loss of a FOLR2^+^ macrophage‐centered homeostatic multicellular module and the emergence of an NLRP3^+^ macrophage‐driven inflammatory network represents a fundamental feature of multicellular remodeling in AAA.

### 3.4 Transcriptional Characterization and Multi‐Cohort Bulk Transcriptomic Validation Confirm M08_Mph_NLRP3 as an AAA‐Enriched Inflammatory Macrophage Population

Having established the disease‐enriched cellular and multicellular network context of M08_Mph_NLRP3 and M09_Mph_FOLR2, we next sought to characterize their transcriptional programs in detail and validate their disease relevance in independent bulk transcriptomic cohorts. We applied Pseudo‐bulk DESeq2 analysis comparisons between AAA and control groups, which uncovered substantial transcriptional variation across cell subsets (Figure 5A). In M09_Mph_FOLR2, AAA was associated with significant downregulation of canonical homeostatic markers including FOLR2, LYVE1, and TIMD4, alongside upregulation of inflammatory mediators, indicating functional reprogramming of tissue‐resident macrophages in the aneurysmal microenvironment. In M08_Mph_NLRP3, AAA marker gene signature was dominated by core inflammasome components including NLRP3, IL1B, CASP1, and CXCL5, consistent with active NLRP3 inflammasome assembly and downstream cytokine release. Of note, M08_Mph_NLRP3 was absent in control samples and excluded from within‐subtype pseudo‐bulk AAA‐versus‐control testing. Genes including NLRP3, IL1B, CASP1, and CXCL5 are its cluster‐specific markers, identified by comparing M08_Mph_NLRP3 against other myeloid subtypes. We further employed GSEA to clarify distinct biological roles for these two macrophage subtypes (Figure 5B). M09_Mph_FOLR2 was enriched in pathways related to defense response to organisms and positive regulation of cytokine production in the control direction, reflecting its homeostatic immune surveillance role. M08_Mph_NLRP3 showed strong enrichment in DNA‐templated replication, negative regulation of viral processes, and cell‐mediated immunity pathways in the AAA direction, alongside secondary alcohol metabolic processes, collectively underscoring its pro‐inflammatory and metabolically activated state. Volcano plots for the remaining myeloid subtypes are presented in Figure S4.

**FIGURE 5:**
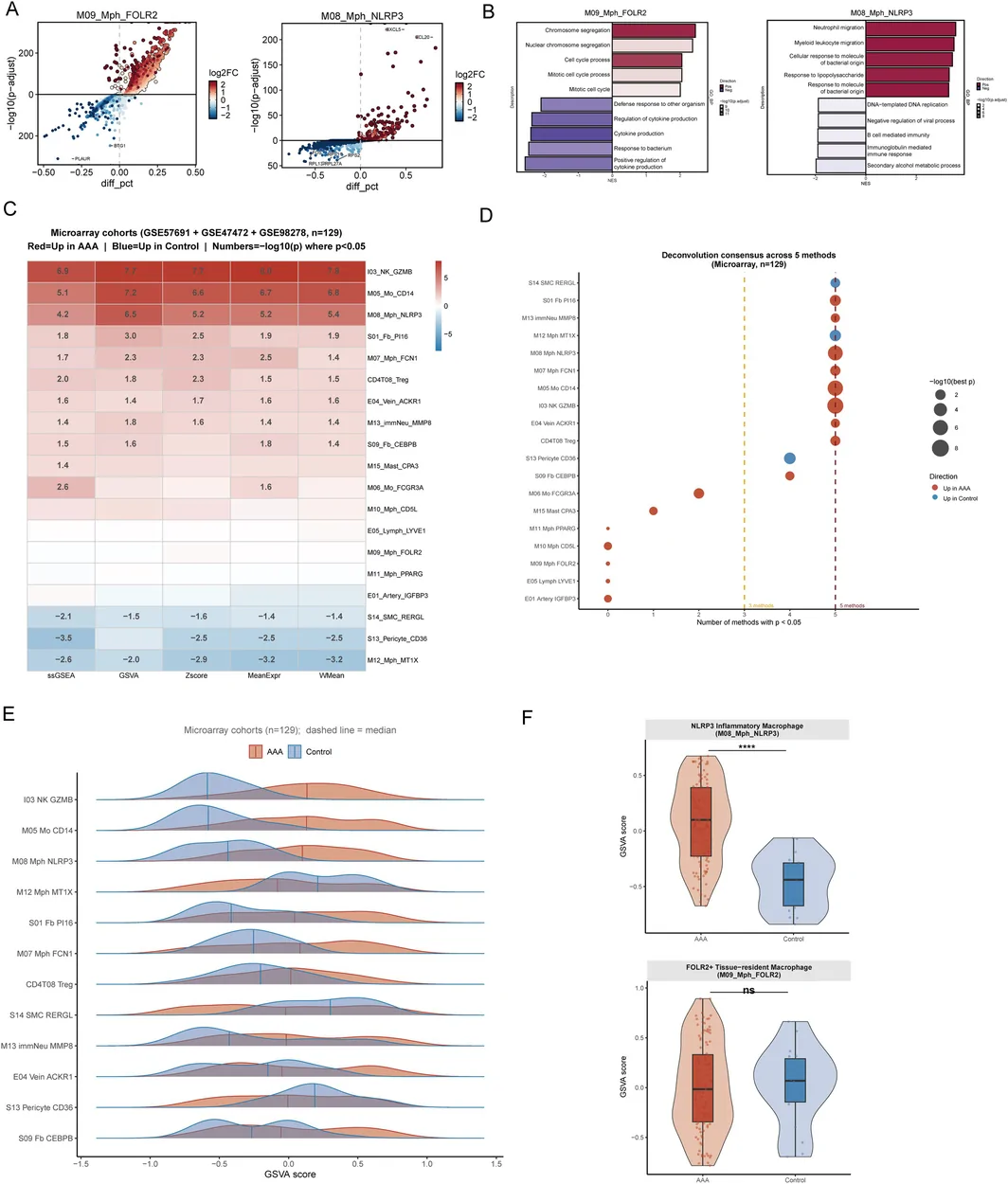
Validation of key cell subset signatures and functional enrichment in AAA using independent public microarray cohorts. (A) Volcano plot visualizing differentially expressed genes. (B) enrichment analyses in M09_Mph_FOLR2 and M08_Mph_NLRP3 populations. (C, D) Heatmap and corresponding statistical summary of gene expression profiles in the integrated microarray cohorts (GSE57691, GSE47472, and GSE98278; *n* = 129). (E) Distribution of GSVA scores in AAA and control groups from the microarray cohorts (*n* = 129). (F) GSVA score comparison of representative cell subset signatures between AAA and control groups (*p < 0.05, **p < 0.01, ***p < 0.001, ****p < 0.0001).

To validate these single‐cell‐derived findings in a larger and independent sample context, we performed signature‐based deconvolution of three publicly available microarray cohorts (GSE57691, GSE47472, and GSE98278; total *n* = 129, comprising 111 AAA and 18 controls) (Figure S5A,B). Five complementary enrichment approaches were applied—ssGSEA, GSVA, Zscore, MeanExpr, and WMean—to ensure robustness of the findings. Across all 19 cell types tested, M08_Mph_NLRP3 emerged as one of the most consistently and significantly elevated populations in AAA, reaching statistical significance in all five methods (consensus −log10 (best *p*) = 4.2) (Figure 5C). Additional cell types significantly elevated in AAA across multiple methods included I03_NK_GZMB, M05_Mo_CD14, M07_Mph_FCN1, S01_Fb_PI16, and CD4T08_Treg, while M12_Mph_MT1X and S14_SMC_RERGL signatures showed higher estimated abundance in controls across multiple deconvolution methods (Figure 5C,D, Figure S6A). Visualization of GSVA score distributions across all 129 samples confirmed a consistent rightward shift for M08_Mph_NLRP3 in AAA relative to controls, with the separation being among the most pronounced of all signatures examined (Figure 5E). Direct comparison of GSVA scores between AAA and control groups demonstrated highly significant elevation of the M08_Mph_NLRP3 signature in AAA (*p* < 0.0001), whereas the M09_Mph_FOLR2 signature did not reach statistical significance (*p* > 0.05) (Figure 5F). The latter finding is attributable to the technical limitation that FOLR2^+^ tissue‐resident macrophage marker genes, including C1QA, MRC1, and CD163, are broadly shared with other macrophage subpopulations, rendering their specific signal indistinguishable at the bulk transcriptome level. The consistent elevation of the M08_Mph_NLRP3 populations across five complementary enrichment approaches—though these methods are algorithmically related as rank‐and mean‐based enrichment approaches rather than truly independent orthogonal techniques—nevertheless reinforces the robustness of the signature against methodological choices. The repeated detection across three separate cohorts further supports the reproducibility of this finding, while acknowledging that these approaches share underlying assumptions and should be interpreted as convergent evidence rather than fully independent validation. (Figure S6B).

### 3.5 Pro‐Inflammatory Stimulation Induces Pathological Macrophage Activation In Vitro AAA Model

To generate an inflammatory macrophage phenotype associated with the transcriptional features observed in the single‐cell analysis. THP‐1 cells were differentiated into macrophage‐like phenotypes with PMA and then stimulated with OX‐LDL, IL‐1β, and LPS alone or in combination. RT‐qPCR and Western Blot analyses were performed to detect the transcriptional and protein expression levels of key markers. According to the WB results, we found that after stimulation with OX‐LDL, pro‐IL‐1β and LPS, the homeostatic marker FOLR2 was significantly downregulated, and the downregulation was most obvious in the combined drug stimulation group. In contrast, after drug stimulation, the expression levels of pathological markers, including NLRP3, pro‐IL‐1β, IL‐18, and Caspase‐1 of the inflammasome axis, as well as MMP8 and MMP9 related to matrix degradation, were increased, with the most significant upregulation in the combined stimulation group (Figure 6A). RT‐qPCR results were consistent with WB findings, confirming that the combined stimulation significantly upregulated the mRNA expression of NLRP3, IL‐1β, IL‐18, MMP8, and MMP9, and prominently downregulated the mRNA expression of FOLR2 (Figure 6B–D).

**FIGURE 6:**
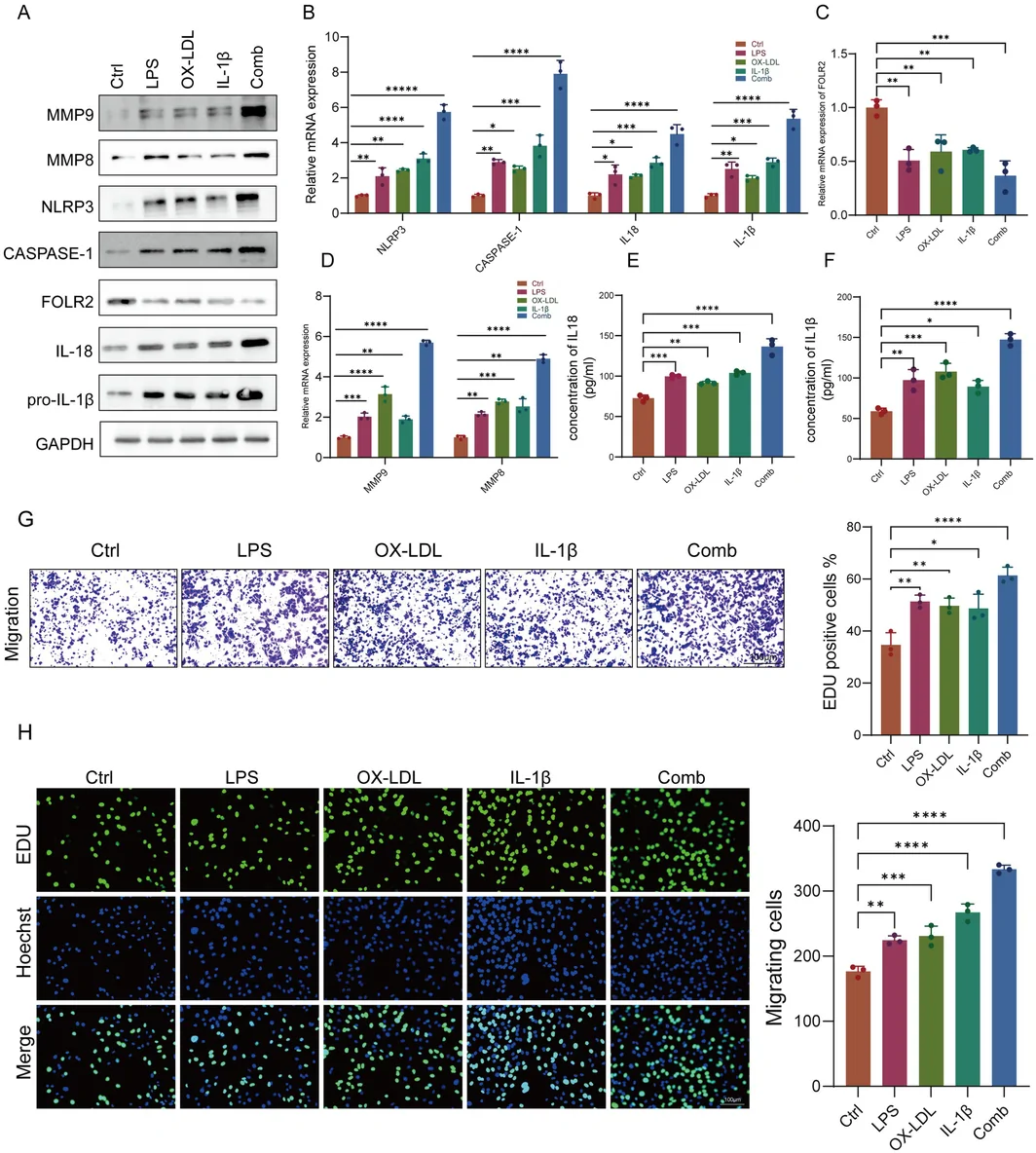
Pro‐inflammatory stimulation induces pathological macrophage activation in vitro AAA model (A–D) Protein and mRNA expression of MMP9, MMP8, NLRP3, Caspase‐1, FOLR2, IL‐18, pro‐IL‐1β in macrophages with different stimulation. (E, F) The concentrations of IL18, IL‐1β in the cell supernatants were measured by ELISAs. (G) Transwell migration assay. (H) EdU proliferation assay. All experiments were performed with three independent biological replicates (*p < 0.05, **p < 0.01, ***p < 0.001, ****p < 0.0001).

Moreover, ELISA results showed that the levels of IL‐1β and IL‐18 were significantly increased following treatment with LPS, OX‐LDL, or IL‐1β compared with the control group, with the highest expression observed in the combined treatment group (Figure 6E,F). To further investigate the proliferating ability, we conducted EdU and Transwell migration assays. Our results demonstrated that stimulation with LPS, OX‐LDL, or IL‐1β significantly promoted macrophage proliferation, and this effect was most prominent in the combined stimulation group (Figure 6G,H). These results confirmed that the established in vitro inflammatory model successfully illustrated the characteristic changes of homeostatic and pathological macrophage markers observed in human AAA tissues, validating the relevance of this model for investigating AAA‐associated macrophage remodeling.

### 3.6 ω‐3 PUFA Reverses the Pathological Phenotype of AAA

To confirm the role of NLRP3 inflammasome activation in mediating the pathological phenotype of AAA‐like macrophages, we treated THP‐1‐derived macrophages with ω‐3 PUFA and MCC950 under combined LPS + IL‐1β stimulation.

First, RT‐qPCR results showed that, similar to MCC950, ω‐3 PUFA markedly suppressed the transcriptional upregulation of caspase‐1, IL‐1β, IL‐18, and GSDMD induced by combined stimulation. (Figure 7A–D).

**FIGURE 7:**
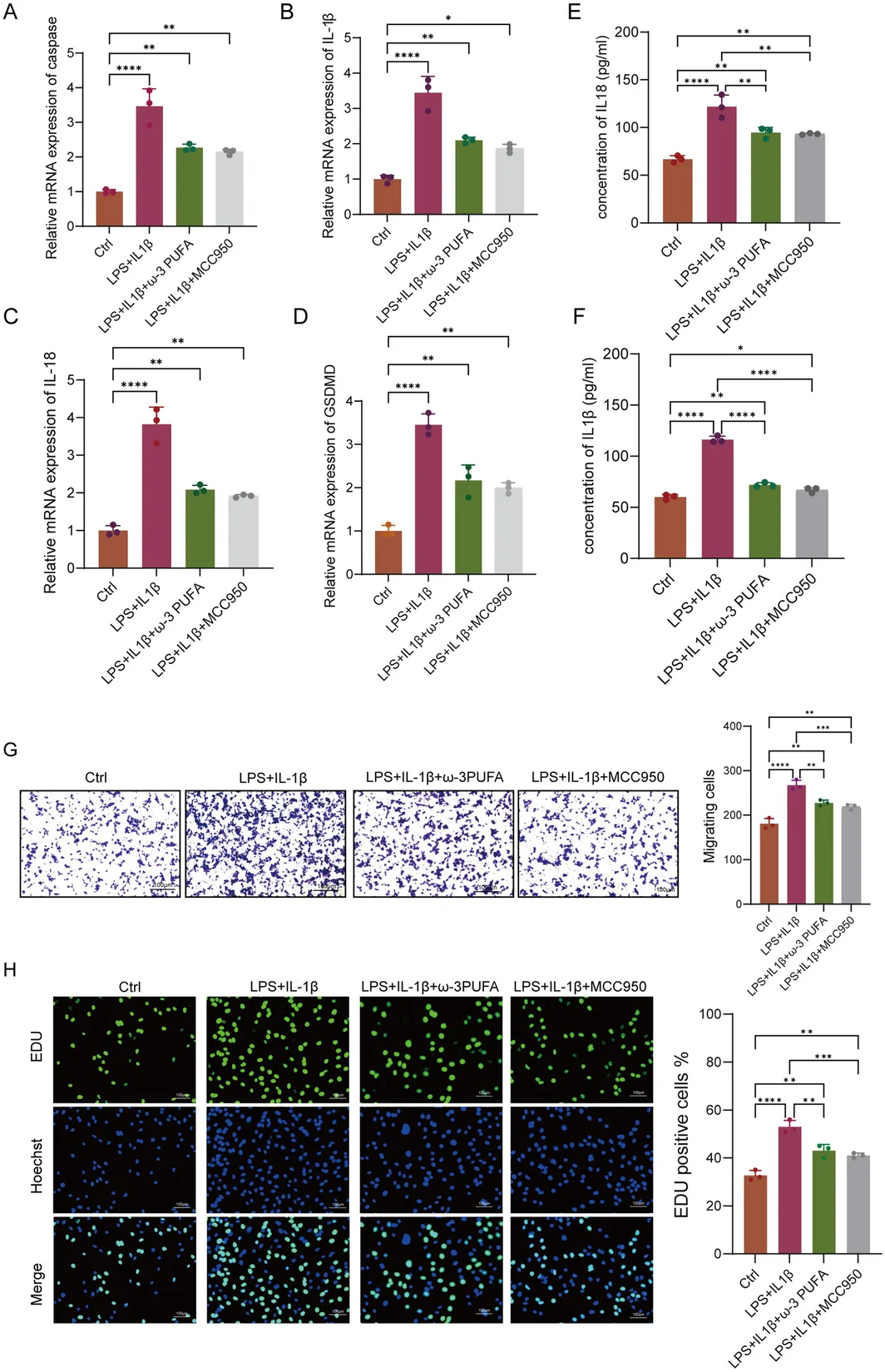
ω‐3 PUFA reverses the pathological phenotype of AAA. (A–D) Relative mRNA expression of caspase‐1, IL‐1β, IL‐18, and GSDMD. (E, F) ELISA analysis of IL‐18 and IL‐1β secretion. (G) Transwell migration assay. (H) EdU proliferation assay. All experiments were performed with three independent biological replicates (*p < 0.05, **p < 0.01, ***p < 0.001, ****p < 0.0001).

Furthermore, ELISA assays confirmed that the elevated secretion of mature IL‐1β and IL‐18 in the culture supernatant upon LPS + IL‐1β stimulation was significantly attenuated by ω‐3 PUFA and MCC950 (Figure 7E,F). Functionally, Transwell migration assays showed that, similar to MCC950, ω‐3 PUFA treatment partially reversed the enhanced migratory capacity of macrophages under inflammatory stimulation (Figure 7G). Similarly, EdU proliferation assays revealed that, similar to MCC950, ω‐3 PUFA treatment significantly reduced the increased proportion of proliferating macrophages observed in the LPS + IL‐1β group (Figure 7H).

Collectively, these results indicate that ω‐3 PUFA attenuated inflammasome‐associated inflammatory signaling and pathological macrophage phenotypes under the experimental conditions used.

## 4 Discussion

In this research, we combined single‐cell transcriptomic data from three AAA datasets and constructed a comprehensive multi‐lineage cellular atlas comprising 64,258 cells and 50 distinct subtypes. We identified a reciprocal shift between homeostatic FOLR2^+^ tissue‐resident macrophages and disease‐enriched NLRP3^+^ inflammatory macrophages as a defining feature of AAA pathology. Notably, we confirmed increased proportions of M08_Mph_NLRP3 using five complementary enrichment approaches in a combined cohort of 129 samples. This solidifies the subset as a reliable and reproducible hallmark of AAA.

Our observation that FOLR2‐positive resident macrophage populations decline within AAA lesions aligns with previous studies in multiple tissues (Liu et al. 2025). FOLR2^+^ macrophages, characterized by high expression of LYVE1, TIMD4, and C1QA, have been shown to play critical roles in extracellular matrix remodeling, efferocytosis, and local immune tolerance (Bugatti et al. 2022; Cohen et al. 2024; Dick et al. 2022; Nalio Ramos et al. 2022; Zhang et al. 2023; Zhao et al. 2024). Previous studies have demonstrated that depletion of these resident macrophage subsets strongly correlates with atherosclerotic progression and plaque instability within the vascular system (Örd et al. 2025). Our findings extend this concept to AAA, suggesting that the progressive depletion of the FOLR2^+^ macrophage population may represent an early event in the breakdown of aortic wall homeostasis, creating a permissive environment for subsequent inflammatory escalation. We observed that M09_Mph_FOLR2 acts as the primary constituent of DM03, a multicellular module enriched in control samples that colocalizes with arterial endothelial cells and structural stromal cells. This reinforces our conclusion that such macrophages actively sustain intact vascular wall structure, instead of merely functioning as inert immune sentinel cells.

The emergence of M08_Mph_NLRP3 as an AAA‐enriched inflammatory macrophage population is a central finding of our work. The NLRP3 inflammasome is an immune sensor that responds to cholesterol crystals, reactive oxygen species, and extracellular matrix degradation products—all of which are abundantly present in the AAA microenvironment (Grebe et al. 2018; Takahashi 2021). Activation of the NLRP3 inflammasome may promote proteolytic processing and secretion of IL‐1β and IL‐18, potent pro‐inflammatory cytokines that amplify vascular inflammation, promote SMC apoptosis, and accelerate extracellular matrix degradation through induction of matrix metalloproteinases (Fujimura et al. 2023; Grebe et al. 2018; Qian et al. 2025). The transcriptional signature of M08_Mph_NLRP3, dominated by NLRP3, IL1B, CASP1, and CXCL5, is fully consistent with an actively engaged inflammasome program. Our single‐cell data now provide direct cellular‐resolution evidence that NLRP3 inflammasome activation is concentrated in a discrete and identifiable macrophage subpopulation that is entirely absent from healthy aortic tissue, lending precision to the mechanistic understanding of NLRP3‐driven inflammation in AAA.

Our experiments further validated the pathological role of NLRP3^+^ macrophages in AAA. EdU proliferation and Transwell migration assays revealed that pro‐inflammatory stimuli remarkably promoted the migration and proliferation of THP‐1‐derived macrophages. The enhanced mobility and proliferation of inflammatory macrophages would aggravate vascular inflammation and tissue damage, driving AAA progression.

Furthermore, ω‐3 PUFA could effectively reverse the above abnormal cellular functions, along with inhibiting inflammasome activation and inflammatory factor release. These results reveal that NLRP3 inflammasome activation comprehensively mediates the pathological transformation of macrophages in terms of molecular phenotype and biological behavior. Together, these results indicate that NLRP3 inflammasome activation in M08_Mph_NLRP3 macrophages may promote vascular inflammation and extracellular matrix breakdown, supporting the therapeutic potential of targeting NLRP3 for the treatment of AAA.

Beyond the characterization of individual cell subtypes, the application of CoVarNet to AAA data provided a systems‐level perspective on how single‐cell changes translate into altered multicellular coordination programs. The CoVarNet framework defined 12 reference CMs representing conserved multicellular coordination programs (Shi et al. 2025). CM01 is a pan‐tissue homeostatic module anchored by FOLR2^+^ resident macrophages, fibroblasts, lymphatic endothelial cells, and NK cells. Its tendency toward lower activity in AAA indicates disrupted multicellular homeostasis in the aneurysmal aortic wall. By contrast, CM12 is originally enriched in reproductive tissues, where estrogen‐driven inflammatory fibroblast dynamics modulate its activity according to menopausal status. Among all 12 reference co‐expression modules, CM12 was the only one showing statistically significant upregulation in AAA samples. Meanwhile, CM01 displayed a trend toward reduced activity in AAA; this indicates reactivation of this inflammatory multicellular program within the diseased aortic wall, triggered by chronic local inflammation rather than hormonal signals. This interpretation is further reinforced by the complementary Discovery analysis, which independently identified a control‐exclusive module (DM03) centered on FOLR2^+^ macrophages and vascular wall cells, and an AAA‐dominant module (DM04) featuring M08_Mph_NLRP3 as a top‐weighted component. The strong cross‐framework concordance between Recovery and Discovery results—evidenced by the high Spearman correlations between CM01 and DM03 (*r* = 0.70) and between CM12 and DM01/DM04 (*r* = 0.78 and *r* = 0.61, respectively)—reinforces the robustness of these findings and demonstrates that the multicellular remodeling in AAA is a structured and reproducible phenomenon rather than stochastic cellular variation. To our knowledge, this represents the first application of multicellular coordination network analysis to AAA research, and the results suggest that the pathological significance of NLRP3^+^ macrophages extends beyond their intrinsic inflammatory activity to encompass their role as organizers of a broader disease–enriched multicellular network, providing a systems‐level framework that complements and extends conventional single‐cell analyses.

The bulk transcriptomic validation analysis deserves specific discussion with respect to both its strengths and limitations. The use of five complementary enrichment approaches applied to a combined cohort of 129 samples substantially increases the confidence in the M08_Mph_NLRP3 findings, as the consistent elevation of this signature across ssGSEA, GSVA, Zscore, MeanExpr, and WMean methods reduces the likelihood that the result is method‐dependent. Our deconvolution results for M08_Mph_NLRP3 were fully concordant with the single‐cell findings, reinforcing its validity as a robust AAA‐associated signature. However, we observed conflicting trends between deconvolution outputs and single‐cell proportion calculations for signatures like M12_Mph_MT1X. This discrepancy likely reflects the limited specificity of bulk deconvolution for closely related macrophage subpopulations whose marker genes are broadly shared—a known limitation of signature‐based methods when applied to transcriptionally similar cell states. We also found that the M09_Mph_FOLR2 gene signature showed no statistically significant change in bulk deconvolution analysis. We believe this arises because its representative markers C1QA, MRC1, and CD163 are widely shared across various macrophage subsets, not because this biological signal does not exist. From these results, we recognize that single‐cell and bulk RNA sequencing can support one another analytically, and we stress that deconvolution outputs from bulk samples must be analyzed together with single‐cell evidence instead of being assessed separately.

We need to acknowledge multiple drawbacks existing within our present research. First, our pseudo‐bulk differential‐expression analysis uses a relaxed FDR threshold of < 0.2, as our single‐cell dataset contains merely three control samples. This relaxed threshold brings an elevated risk of false positive DEG calls. Future single‐cell studies with larger, better‐balanced case–control cohorts will be required to enable robust per‐gene DE analysis with strict multiple‐testing correction. Of note, the Wilcoxon rank‐sum tests comparing CM activity between AAA and control across all 12 reference CMs were not adjusted for multiple comparisons. The reported nominal *p* value of 0.030 for CM12 should therefore be interpreted with caution and does not guarantee robust statistical significance after accounting for multiple testing.

In addition, given the small sample size of our single‐cell dataset (only three control samples), sensitivity, permutation, or resampling analyses for validating the robustness of CM‐level associations are statistically underpowered and computationally infeasible in the current study. Further independent cohorts with larger sample sizes are required to confirm these multicellular module‐level findings. In addition, aggregated public single‐cell datasets lack some donor‐specific metadata, precluding us from analyzing the distribution of M08_Mph_NLRP3 cells across individual AAA donors. We acknowledge this limitation. In future work, we plan to utilize in‐house clinical specimens and perform immunohistochemistry to further characterize the distribution of this macrophage subset within AAA patient samples. Second, although two of our bulk datasets (GSE57691 and GSE47472) contain both AAA and control samples, we did not perform separate AAA‐versus‐control signature analyses for each individual cohort. Our case–control findings are derived from batch‐corrected pooled‐cohort analysis. Furthermore, limited sample size prevented us from building a pseudo‐bulk design matrix incorporating both cohort and experimental condition. Independent single‐cohort validation and future large‐sample‐size analyses accounting for both cohort and disease‐group variables will further strengthen our conclusions. Third, the absence of spatial transcriptomic data in our study means that the spatial localization of M08_Mph_NLRP3 within the aortic wall layers—and the physical proximity of the multicellular modules identified by CoVarNet—remain to be directly demonstrated. Fourth, while our CoVarNet Discovery analysis was performed with careful attention to NMF rank selection and stability metrics, the relatively small sample size (*n* = 15) limits the granularity of multicellular module resolution. Moreover, it should be noted that the five complementary enrichment approaches employed for bulk transcriptomic validation are algorithmically related variants that rely on rank‐based or mean‐based enrichment calculations. They do not constitute truly independent orthogonal validation strategies. Therefore, the cross‐method consistency should be interpreted as evidence of robustness against parameter choices within this class of algorithms, rather than as independent technical validation. Future studies incorporating orthogonal approaches would be valuable to further substantiate the cellular findings. Fifth, although we have introduced the MCC950 in this study, validation at the western‐blot protein level is lacking. Further biochemical experiments at the protein level will be performed in follow‐up mechanistic studies to consolidate these findings. Finally, THP‐1‐derived macrophages were used for in vitro assays instead of primary human aortic macrophages. Both triple‐stimulus (LPS/ox‐LDL/IL‐1β) and simplified dual‐stimulus (LPS + IL‐1β) models only recapitulate partial transcriptional features of AAA‐related macrophages and cannot fully mimic the complex in vivo AAA microenvironment. Ox‐LDL was excluded in ω‐3 PUFA mechanistic tests to specifically evaluate NLRP3‐inflammasome‐dependent signals; future work will reintroduce lipid stress from ox‐LDL for more complete evidence. All functional assays were performed in an in vitro THP‐1 cell model, without supporting in vivo animal experimental data. Subsequent in vivo intervention studies using AAA animal models are needed to validate the systemic protective effects of ω‐3 PUFA.

Currently, the upstream drivers of M08_Mph_NLRP3 activation in AAA remain incompletely defined. Nevertheless, multiple microenvironmental cues are likely involved: the AAA microenvironment is characterized by persistent inflammatory infiltration, oxidative stress, and mechanical stretch—all established danger signals for NLRP3 inflammasome priming and activation (Maruyama et al. 2019); the lipid‐rich milieu of the aortic wall—particularly the accumulation of oxidized low‐density lipoprotein and cholesterol crystals—is known to activate the NLRP3 inflammasome via lysosomal damage and K^+^ efflux (Bai et al. 2021; Stitham et al. 2020); and DAMPs released from extracellular matrix remodeling, such as biglycan and tenascin‐C, have been reported to engage TLR2/4 and promote NLRP3 activation in macrophages (Lambert et al. 2020; Duewell et al. 2010). These microenvironmental signals likely converge to drive the enrichment of the M08_Mph_NLRP3 subset in AAA. However, the relative contribution of each signal warrants systematic investigation in future studies.

Collectively, we mapped the macrophage landscape at varied resolution levels in AAA, revealing that the reciprocal loss of homeostatic FOLR2^+^ macrophages and emergence of NLRP3^+^ inflammatory macrophages represent a fundamental axis of cellular remodeling in this disease. M08_Mph_NLRP3 has been confirmed as the core hub of the AAA‐exclusive multicellular inflammatory network via diverse analytical tools and external sample sets. This discovery makes the NLRP3 inflammasome a promising potential cellular target. Our outcomes also lay cellular and systematic groundwork for developing NLRP3‐directed treatments and macrophage‐related biomarkers to monitor AAA progression.

## 5 Conclusions

In conclusion, our study identified a core cellular remodeling paradigm in AAA: the loss of homeostatic FOLR2^+^ macrophages and the emergence of NLRP3^+^ inflammatory macrophages (M08_Mph_NLRP3). M08_Mph_NLRP3 served as the central node of a disease‐enriched inflammatory network. In vitro experiments confirmed that NLRP3 inflammasome activation drives AAA‐related inflammation and matrix degradation, which can be alleviated by ω‐3 PUFA. These findings highlight NLRP3^+^ macrophages as a promising potential cellular target for AAA.

## Author Contributions

**Yuqi Wang:** conceptualization, investigation, funding acquisition, writing – original draft, writing – review and editing, visualization, methodology, data curation. **Hongrui Pan:** investigation, methodology, software. **Hechen Rong:** methodology, software, data curation. **Yu Zeng:** investigation, software, project administration, validation. **Yinghong Li:** formal analysis, supervision. **Xiang Li:** formal analysis, validation. **Zhian Liang:** visualization. **Jiaxin Wang:** validation. **Lijian Fang:** validation. **Peng Li:** project administration. **Xiangchen Dai:** funding acquisition, project administration, writing – review and editing.

## Funding

This study was funded by the National Natural Science Foundation of China (no. 82241207 and 82070489). Tianjin Key Medical Discipline Construction Project (no. TJYXZDXK‐3‐001B), Tianjin Medical University Integrated Chinese and Western Medicine Discipline Enhancement Program (no. 2024XKZXY01), and Tianjin Science and Technology Popularization Project (no. 25KPXCRC00160).

## Ethics Statement

The authors have nothing to report.

## Consent

The authors have nothing to report.

## Conflicts of Interest

The authors declare no conflicts of interest.

## Supporting information


**Figure S1:** (A) GO enrichment analysis of differentially expressed genes (DEGs) in endothelial cells. (B) GO enrichment analysis of DEGs in B cells. (C) UMAP visualization of endothelial cell subtypes and their molecular signatures. (D) B cell subclustering and marker gene expression. (E) CD4T cell subclustering and marker gene (F) IL C/NK subclustering and marker gene expression.
**Figure S2:** (A) Box plots comparing activity scores of 10 cellular modules (CM02–CM11) in AAA versus control. (B) Multicellular coordination networks of cellular modules (CM02–CM11). (C) Cell–cell communication network of immune cells in AAA.
**Figure S3:** (A) NMF rank selection based on multiple evaluation metrics. The heatmap shows the weight of cell‐type‐specific gene signatures (rows) across six NMF metagene programs (columns, DM01–DM06). (B) The heatmap shows the weight of cell‐type‐specific gene signatures (rows) across six NMF metagene programs (columns, DM01–DM06). (C) Cell–cell communication network in AAA.
**Figure S4:** (A) Volcano plots for the different myeloid subtypes.
**Figure S5:** (A, B) Data preprocessing for the integrated AAA transcriptome dataset.
**Figure S6:** (A) GSVA score comparison of different cell subset signatures between AAA and control groups. (B) correlation analysis all five deconvolution approaches for the significant signatures
**Table S1:** Antibody.
**Table S2:** Primer sequences.

## Data Availability

The datasets used during the present study are available from the corresponding authors upon reasonable request.
