# Supplementary material for: NLRP3+ Macrophages: Central Inflammatory Nodes of Abdominal Aortic Aneurysm and Potential Cellular Target of Omega‐3 Polyunsaturated Fatty Acids
Source: Food Sci Nutr. 2026 Nov 10;14(11):e72414. doi: 10.1002/fsn3.72414 (PMC13752396; doi:10.1002/fsn3.72414)
Supplement: Supplementary file 1 [file FSN3-14-e72414-s001.docx]

**Supplementary Figure 1**

**(A)** GO enrichment analysis of differentially expressed genes (DEGs) in endothelial cells. **(B)** GO enrichment analysis of DEGs in B cells. **(C)** UMAP visualization of endothelial cell subtypes and their molecular signatures. **(D)** B cell subclustering and marker gene expression. **(E)** CD4T cell subclustering and marker gene **(F)** IL C/NK subclustering and marker gene expression.

**Supplementary Figure 2**

(A) Box plots comparing activity scores of 10 cellular modules (CM02–CM11) in AAA vs. control. (B) Multicellular coordination networks of cellular modules (CM02–CM11). (C)Cell-cell communication network of immune cells in AAA.

**Supplementary Figure 3**

(A)NMF rank selection based on multiple evaluation metrics. The heatmap shows the weight of cell-type-specific gene signatures (rows) across six NMF metagene programs (columns, DM01–DM06). (B) The heatmap shows the weight of cell-type-specific gene signatures (rows) across six NMF metagene programs (columns, DM01–DM06). (C) cell-cell communication network in AAA.

**Supplementary Figure 4**

**(A)**Volcano plots for the different myeloid subtypes

**Supplementary Figure 5**

**(A-B)** Data preprocessing for the integrated AAA transcriptome dataset.

**Supplementary Figure 6 (A)**GSVA score comparison of different cell subset signatures between AAA and control groups. **(B)** correlation analysis all five deconvolution approaches for the significant signatures

**Supplementary Table1** Antibody

| FOLR2 | Proteintech 31264-1-AP |
| --- | --- |
| MMP9 | Proteintech 10375-2-AP |
| MMP8 | Proteintech 17874-1-AP |
| NLRP3 | Proteintech 30109-1-AP |
| Caspase-1 | Proteintech 22915-1-AP |
| IL-18 | Proteintech 10663-1-AP |
| Pro-IL-1β | Proteintech 29530-1-AP |
| GAPDH | Proteintech 60004-1-Ig |

**Supplementary Table2** primer sequences

| gene | Forward primer | Reverse primer |
| --- | --- | --- |
| β-actin | GATCATTGCTCCTCCTGAGC | ACTCCTGCTTGCTGATCCAC |
| NLRP3 | GATCTTCGCTGCGATCAACAG | CGTGCATTATCTGAACCCCAC |
| Caspase-1 | AGCACGTTCCTGGTGTTCAT | TTGGGCAGTTCTTGGTATTCA |
| IL-18 | CCCCGGACCATATTTATT | GTTATCAGGAGGATTCATTTC |
| IL-1β | ATGATGGCTTATTACAGTGGCAA | GTCGGAGATTCGTAGCTGGA |
| FOLR2 | CCTCTGGAGTCACTCATACAAGG | CCTGGGCTGAATCAAACCAC |
| MMP9 | TTGACAGCGACAAGAAGTGG | GCCATTCACGTCGTCCTTAT |
| MMP8 | TGCTCTTACTCCATGTGCAGA | TCCAGGTAGTCCTGAACAGTTT |
| GSDMD | GTGTGTCAACCTGTCTATCAAGG | CATGGCATCGTAGAAGTGGAAG |
